# Long-Term Efficacy and Safety of Hizentra® in Patients with Primary Immunodeficiency in Japan, Europe, and the United States: a Review of 7 Phase 3 Trials

**DOI:** 10.1007/s10875-018-0560-5

**Published:** 2018-11-10

**Authors:** Stephen Jolles, Mikhail A. Rojavin, John-Philip Lawo, Robert Nelson, Richard L. Wasserman, Michael Borte, Michael A. Tortorici, Kohsuke Imai, Hirokazu Kanegane

**Affiliations:** 1 Immunodeficiency Centre for Wales, University Hospital of Wales, Cardiff, UK; 2 CSL Behring LLC, King of Prussia, PA USA; 3 CSL Behring GmbH, Marburg, Germany; 4 School of Medicine and the Melvin and Bren Simon Cancer Center, Indiana University, Indianapolis, IN USA; 5 Medical City Children’s Hospital, Dallas, TX USA; 6 Hospital St. Georg GmbH Leipzig, Academic Teaching Hospital of the University of Leipzig, Leipzig, Germany; 7 Department of Community Pediatrics, Perinatal and Maternal Medicine, Tokyo Medical and Dental University (TMDU), Tokyo, Japan; 8 Department of Child Health and Development, Graduate School of Medical and Dental Sciences, Tokyo Medical and Dental University (TMDU), Tokyo, Japan

**Keywords:** Immunoglobulin G replacement therapy, primary antibody deficiencies, primary immunodeficiencies, IVIG, SCIG

## Abstract

Many patients with primary immunodeficiency (PID) require immunoglobulin G (IgG) replacement therapy, delivered as intravenous IgG (IVIG) or subcutaneous IgG (SCIG). We aim to identify trends in efficacy and safety that would not be evident in individual studies of small patient numbers. Seven open-label, Phase 3, prospective, multicenter studies of the efficacy and safety of Hizentra® (a SCIG), conducted in Japan, Europe, and the US were summarized. Overall, 125 unique patients received 15,013 weekly infusions during a total observation period of 250.9 patient-years. Mean weekly doses of Hizentra® were 83.22–221.3 mg/kg body weight; infusion rates per patient (total body rate) were 25.2–49.3 mL/h across studies. The rates of infections and serious bacterial infections were 3.10 and 0.03 events per patient/year, respectively. Annualized rates of days hospitalized due to infection, out of work/school, and prophylactic antibiotic use were 0.95, 5.14, and 36.78 per patient, respectively. For the equivalent monthly dose, weekly Hizentra® SCIG administration resulted in expectedly-increased serum IgG trough levels in patients switching from IVIG, and maintained levels in patients switching from previous SCIG. Adverse events (AEs) totaled 5039 (events/infusion 0.094–0.773), almost all of which were mild/moderate. Three thousand one hundred ninety-seven were considered treatment-related, the most common of which were injection site reactions (2919 events; 0.001–0.592 AEs per infusion). Systemic AEs were very uncommon. The results from these seven studies indicate that Hizentra® therapy was both efficacious and well tolerated during long-term treatment. This is particularly important in patients with PID, who may require lifelong IgG replacement therapy.

**Electronic supplementary material:**

The online version of this article (10.1007/s10875-018-0560-5) contains supplementary material, which is available to authorized users.

## Introduction

The majority of patients with primary immunodeficiency (PID), including common variable immunodeficiency (CVID) and X-linked agammaglobulinemia (XLA), require immunoglobulin G (IgG) replacement therapy [1–3]. Intravenous IgG (IVIG) and subcutaneous IgG (SCIG) are two options for the delivery of this treatment [4].

SCIG and IVIG are equally effective [5, 6]; however, SCIG does not require venous access, and is associated with improved quality of life for patients [7], more stable serum IgG level profiles [8], potential reductions in “wear-off effect” [9], lower incidence of systemic adverse events (AEs) [6], and reduced cost [10], compared with IVIG. SCIG delivery also permits the patient flexibility with their treatment schedule, and enables home-based self-administration for many patients [1, 7].

Hizentra® (CSL Behring, King of Prussia, PA, USA) was the first 20% liquid IgG product approved for SCIG administration. The high IgG concentration permits administration of smaller volumes, while the relatively low viscosity facilitates high infusion rates [11, 12]. Five published clinical trials provide evidence that Hizentra® is efficacious and well tolerated by patients with PID [12–15]. Herein, we summarize results from those trials and two additional unpublished extension studies to further define long-term efficacy and safety in a global context. This integrated summary sought to identify trends in efficacy and safety that might not be evident in individual trials of small numbers of patients, as often occurs with clinical trials in PID.

## Methods

### Patients and Study Designs

Data were reviewed from seven open-label, Phase 3, prospective, multicenter studies as follows: (1) Japan (pivotal [NCT01199705] [15], follow-up [NCT01458171], and extension [NCT01461018] studies; performed September 2010–July 2014); (2) Europe (pivotal [NCT00542997] [13] and extension [NCT00751621] studies [14]; performed September 2007–December 2011), and (3) the US (pivotal [NCT00419341] [12] and extension [NCT00719680] studies [14]; performed November 2006–June 2010) were included in the analysis.

Methods used in five of these studies were published previously [12–15]. Included in Supplementary Material are previously unpublished aspects of the study design, methods, and results including longer term follow-up results from the pivotal study from Japan [15].

Patients included in the analysis were those who had confirmed PID previously treated with IVIG at 3–4 weekly intervals either for three doses (Japan pivotal study) or 3 months (US pivotal study); the European pivotal study included patients administered IVIG at 3–4 weekly intervals or SCIG at regular weekly intervals, both for at least 6 months. The patient ages ranged from 2 to 75 years (Japan and US pivotal studies), 2 to ≤ 65 years (European pivotal study), and 16–65 (UK sites within the European study).

Major exclusion criteria included the following: (1) newly diagnosed PID (i.e., not having received previous IgG replacement therapy); (2) serious bacterial infection (SBI) at the time of screening or first infusion; (3) lymphoreticular malignancies including chronic lymphocytic leukemia, non-Hodgkin’s lymphoma, or thymoma with immunodeficiency; (4) a positive PCR result at screening for any of the following viral markers: human immunodeficiency virus, hepatitis C virus, or hepatitis B virus.

**Fig. 1:**
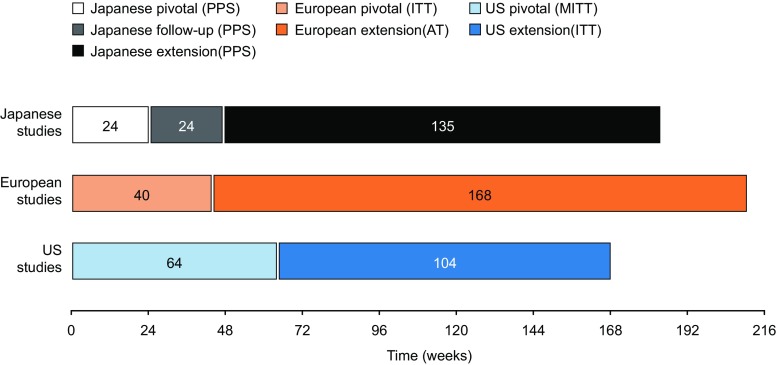
Study duration. AT all treated, ITT intention-to-treat, MITT modified intention-to-treat, PPS per protocol set

The duration of each of the seven studies is shown in Fig. 1. The Japanese pivotal study included a screening period, an IVIG treatment period (three infusions), a 12-week SCIG wash-in/wash-out period, and a 12-week SCIG efficacy period. The European pivotal study included a 12-week wash-in/wash-out period followed by a 28-week efficacy period. The US pivotal study included a 12-week wash-in/wash-out period followed by a 12-month efficacy period. For patient disposition, see Fig. S1.

The majority of doses were administered at home following patient self-infusion or their caregiver’s infusion technique training. During site visits, infusions were performed under the supervision of study site personnel. In the Japanese and European pivotal studies, Hizentra® dosages were equivalent to those received during their previous treatment regimen (either IVIG or alternative SCIG). In the US pivotal study, a conversion factor of 1.53 was used to calculate a monthly SCIG dose bioequivalent to the previous IVIG dose. This conversion factor was based on previous pharmacokinetic studies that showed a dose ratio of SCIG (Hizentra®):IVIG of 1.53 (range 1.26–1.87) led to an area under the curve (AUC) [8] equivalent to IVIG. Subsequent pharmacometric modeling revealed that the total systemic IgG exposure at steady-state remains within the common equivalence range for the AUC ratio (0.8–1.25) with a dose conversion factor of 1.30–1.53 when switching from 4-weekly IVIG to weekly SCIG [16]. In the Japanese and European studies, 1:1 dosing was used, as the regulatory authorities in these areas recognize the equivalence of serum IgG trough levels [11, 17].

In all follow-up and extension studies, the doses used remained the same on a mg/kg basis as in the previous pivotal or follow-up study. Doses in all studies could be adjusted if medically indicated, or if a patient’s weight changed by more than 5% at any time during the study period. In the US extension study, dose adjustment was permitted if an individual’s steady-state IgG trough level resulted in a trough level ratio (ratio of trough on Hizentra® to the last steady-state trough on the pre-study IVIG therapy) that differed by more than 15% from the pre-specified value of 1.29.

### Efficacy and Safety Assessments

The objectives and endpoints of each study are described in Table S1. Efficacy endpoints included the rate of SBIs (defined as bacterial pneumonia, bacteremia/septicemia, osteomyelitis/septic arthritis, bacterial meningitis, or visceral abscess) [18]; the number of infection episodes (serious and non-serious); serum IgG trough levels; the number of days hospitalized due to infections; the number of days out of work, school, kindergarten, or daycare or parental restriction of normal activities due to infections (hereafter referred to as days out of work/school); and the duration of antibiotic use for infection prophylaxis and treatment.

Safety endpoints included the type, number, rate, severity and treatment-relatedness of any AEs per infusion and per patient, and local tolerability of subcutaneous Hizentra® administration. AEs were coded using the Medical Dictionary for Regulatory Activities (MedDRA) version 14.1 in the Japanese and European extension studies, version 12.0 in the European pivotal study, version 11.0 in the US pivotal study, and version 13.1 in the US extension study.

The initial US Hizentra® study procedures included injection site reaction (ISR) evaluations by the patient and the physician, using a variety of methods and multiple mandatory time points during and after infusion. Most ISRs in the US pivotal study were transient, mild, expected, and spontaneously resolving, with approximately 50% of ISRs resolving within 24 h post infusion. Subsequently, less stringent patient/caregiver evaluations (at 24 h [US extension study], at 24–72 h [European pivotal, Japan pivotal, and follow-up study], or at an unspecified time point [European and Japan extension studies]) were used to assess ISRs.

### Pharmacokinetic Methods

A population pharmacokinetic (PK) analysis was conducted using IgG serum concentration data from the clinical trials described above and in Table S1. The development of the population PK model utilized previously-published models as the basis for this analysis [19]. Initial reference models were based on a standard two-compartment PK model, with subcutaneous absorption modeled as a first-order process, and body weight exponents on the key parameters central volume of distribution (V2) and clearance (CL). Endogenous IgG levels of 1.5 g/L and 4 g/L were tested. Covariate testing was performed on body weight, ethnicity, and age. The model was evaluated based on standard diagnostics.

### Statistical Methods

Definitions of each data set are summarized in Table S2 using descriptive statistics. Efficacy results were analyzed in the all-treated (AT), intention-to-treat (ITT), modified ITT, or per-protocol data sets, whereas safety was evaluated in the AT or ITT populations.

Annualized rates were calculated by dividing the number of episodes observed (y) by the total exposure days (T) and multiplying by 365. Upper limits of 99% confidence intervals (CIs) were calculated using the following formula, where T and y are defined as above: (365/T) × (0.5 × *χ*^2^_(0.99,2 × y + 2)_). The annualized rate of SBI was compared with the target rate of <1.0 SBI per patient per year, as recommended by the US Food and Drug Administration (FDA) [18].

Total serum IgG trough levels during Hizentra® therapy were compared with those achieved during the mandatory IVIG treatment periods by calculating the geometric mean ratios (GMR) and respective 90% CIs.

The individual rates of AEs per infusion were calculated for each patient by counting all AEs experienced by one patient and dividing by the total number of Hizentra® infusions administered to this patient. Similar calculations were performed for overall rates of AEs per infusion, using the total number of AEs divided by the total number of infusions.

The analysis of the number of infections per calendar month utilized a logistic regression model based on a Poisson distribution using SAS® PROC GENMOD (SAS Institute Inc., Cary, NC, USA). The possibility of a seasonal effect was assessed based on pairwise differences between months. No adjustment for multiplicity was made in the exploratory analysis of differences between months and the level of significance was set to 5% for each comparison. Seasonal effect was further assessed by fitting a LOESS regression to obtain a reasonable fit of the observed values. Based on this result, a GENMOD model was built to test the model parameters.

Comparisons between the rates of AEs in patients receiving an IVIG product, Privigen® (IgPro10, CSL Behring, King of Prussia, PA, USA) and Hizentra® were performed using the safety data from the Japanese follow-up and extension studies, all European and US studies, and two multicenter studies of Privigen (NCT00168025 and NCT00322556). Specific MedDRA preferred terms for reactions typical for IgG replacement therapy, (injection/infusion site reactions, fatigue, headache, nausea, vomiting, and pyrexia) were identified and searches performed using the MedDRA data collected from each study. Event rates per compound were calculated as total events over the total number of infusion (14,696 for Hizentra® and 1809 for Privigen®). The ratio of rates and its 95% CIs were calculated as outlined in Graham et al. [20].

All analyses were carried out using SAS® software version 9.4 (SAS Institute Inc., Cary, NC, USA).

## Results

### Patients

A total of 125 unique patients in the ITT/AT populations received 15,013 weekly infusions; treatment characteristics are presented in Table 1. Across studies, a total of 43 patients discontinued SCIG for numerous reasons, the most common being withdrawal of consent (*n* = 20), AEs (*n* = 12) and “other” reasons, such as loss to follow-up, termination of study site, non-compliance etc. (*n* = 11).

**Table 1:** Treatment characteristics

Study	Japanese pivotal (PPS)	Japanese follow-up (PPS)	Japanese extension (PPS)	European pivotal (ITT)	European extension (AT)	US pivotal (MITT)	US extension (ITT)
Total number of patients	21	19	17	46	40	38	21
Weeks of enrollment	24	24	135	40	168	64	104
Total SCIG infusions	504	456	2123	1794	5405	2,264^a^	1735
Weekly dose, mg/kg bw, mean (SD)	83.22 (33.15)	97.56 (35.81)	90.31 (31.38)	118.7 (35.51)	115.5 (29.41)	213.2 (77.98)^b^	221.3 (73.38)^b^
Infusion rate, mL/h, mean (SD)	25.2^c^ (6.6)	27.1 (5.6)	27.9 (5.6)	25.1 (9.3)	n.a.	39.1^d^ (13.4)	49.3 (19.8)
Infusion duration, h, mean (SD)	0.98^c^ (0.50)	0.97 (0.42)	1.07 (0.49)	1.27 (0.53)	n.a.	2.31 (1.20)	2.08 (1.16)

*AT* all treated, *bw* body weight, *ITT* intention-to-treat, *MITT* modified intention-to-treat, *n* number of patients, *n.a.* data not available, *PPS* per-protocol set, *SCIG* subcutaneous immunoglobulin, *SD* standard deviation

^a^ITT population

^b^Mean of individual patients’ median weekly doses

^c^During the efficacy period

^d^Mean of individual median infusion rates

In Japan, Europe, and the US, patients were enrolled for up to 183, 208, and 168 consecutive weeks, respectively (Table 1, Fig. 1). The populations testing efficacy included 108 unique patients; who were treated for a total of 91,567 days (250.9 years). Across the PPS populations, 84 unique patients received Hizentra® for a treatment period ≥1 year.

Baseline patient characteristics for each study are presented in Table 2. There were 11, 23, and 10 children and adolescents in the Japanese, European, and US pivotal studies, respectively. There were six patients aged ≥65 years amongst pivotal trials, all from the US pivotal trial.

**Table 2:** Baseline patient characteristics

Study	Japanese pivotal (AT)	Japanese follow-up (AT)	Japanese extension (AT)	European pivotal (AT)	European extension (AT)	US pivotal (ITT)	US extension (AT)
Total number of patients	25	23	22	51	40	49	21
Gender, *n* (%)							
Female	9 (36.0)	9 (39.1)	9 (40.9)	16 (31.4)	12 (30.0)	27 (55.1)	15 (71.4)
Male	16 (64.0)	14 (60.9)	13 (59.1)	35 (68.6)	28 (70.0)	22 (44.9)	6 (28.6)
Age (years)							
Mean (SD)	20.6 (13.32)	20.8 (13.68)	21.6 (14.0)	22.6 (16.0)	21.6 (15.3)	34.4 (20.1)	42.4 (18.5)
Median (range)	18.0 (3–58)	17.0 (4–58)	18.5 (4–59)	18.0 (3–60)	16.0 (4–52)	32.0 (5–72)	42.0 (11–69)
Age group, *n* (%)^a^							
2–11 years	7 (28.0)	6 (26.1)	5 (22.7)	18 (35.3)	15 (37.5)	3 (6.1)	1 (4.8)
12–15 years^b^	4 (16.0)	5 (21.7)	5 (22.7)	5 (9.8)	4 (10.0)	7 (14.3)	1 (4.8)
16–64 years^c^	14 (56.0)	12 (52.2)	12 (54.5)	28 (54.9)	21 (52.5)	33 (67.3)	16 (76.2)
≥65 years	0 (0.0)	0 (0.0)	0 (0.0)	0 (0.0)	0 (0.0)	6 (12.2)	3 (14.3)
Body mass index (kg/m^2^)							
Mean (SD)	18.9 (3.68)	18.9 (3.19)	19.2 (3.1)	20.6 (4.7)	20.5 (4.7)	n.a.	26.4 (6.5)
Median (range)	18.2 (15–33)	18.4 (15–30)	18.8 (15–29)	20.2 (12–32)	20.6 (14–31)	n.a.	26.2 (18–43)
Primary disease, *n* (%)							
CVID	10 (40.0)	10 (43.5)	10 (45.5)	30 (58.8)	23 (57.5)	46 (93.9)	21 (100.0)
XLA	13 (52.0)	11 (47.8)	10 (45.5)	20 (39.2)	16 (40.0)	3 (6.1)	0 (0.0)
ARAG	1 (4.0)	1 (4.4)	1 (4.5)	1 (1.9)	1 (2.5)	0 (0.0)	0 (0.0)

*ARAG* autosomal recessive agammaglobulinemia, *AT* all treated, *CVID* common variable immune deficiency, *FAS* full analysis set, *ITT* intention-to-treat, *n* number of patients, *n.a*. data not available, *SD* standard deviation, *XLA* X-linked agammaglobulinemia.

^a^There were no patients <2 years of age

^b^In the Japanese pivotal, follow-up, and extension studies, this group included patients aged 12–16 years

^c^In the Japanese pivotal, follow-up, and extension studies, this group included patients aged 17–64 years

In the Japanese and European pivotal studies, the proportion of patients with XLA was relatively high; therefore, male participants outnumbered females. The Japanese pivotal study included a female patient with a rare extremely skewed X-chromosome inactivation leading to XLA whose diagnosis had been previously confirmed [21]. Most patients in the US pivotal study had CVID, and the ratio of men to women was more equal.

### Study Drug Administration

Mean weekly doses of Hizentra® ranged from 83.22 mg/kg (standard deviation [SD], 33.15) in the Japanese pivotal study to 221.3 mg/kg (SD, 73.38) in the US extension study (Table 1). Mean infusion rates within studies ranged from 25.2 mL/h in the Japanese pivotal study to 49.3 mL/h in the US extension study (Table 1), while mean (SD) infusion duration ranged from 0.98 (0.50) h in the Japanese follow-up study to 2.31 (1.20) h in the US pivotal study (Table 1).

### Efficacy

**Fig. 2:**
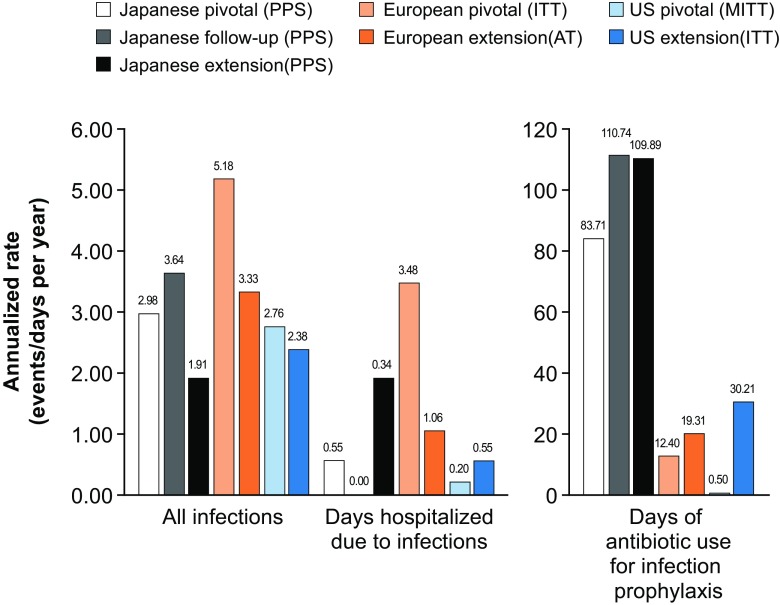
Summary of infections, days hospitalized due to infection, and use of antibiotics for infection prophylaxis. Annualized rates of infections and antibiotic use for prophylaxis are calculated from the number of study days; annualized days hospitalized due to infection are calculated from the number of patient diary days. AT all treated, ITT intention-to-treat, MITT modified intention-to-treat, PPS per protocol set

Overall, there were seven SBIs in the combined studies, and the annualized rate of SBIs in the combined studies was 0.03 (upper 99% CI limit 0.064, Table S3). There were no SBIs in the Japanese studies, the European pivotal study, and the US pivotal study (annualized rates 0). For the US pivotal study, this meant that its primary objective of an annualized SBI rate of <1 per patient was met. A total of 778 infections were reported in the combined studies, with an annualized rate of 3.10 (upper 99% CI limit 3.37) events per patient (Table S3). The annualized rate of infections in individual studies ranged from 1.91 to 5.18 (Fig. 2).

**Fig. 3:**
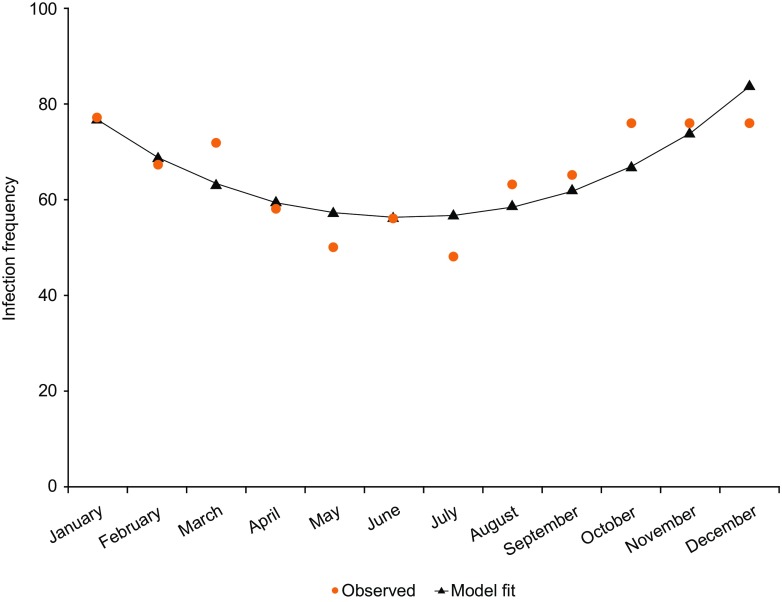
Frequency of infections per calendar month. The frequency of infections per calendar month was analyzed using a logistic regression model based on a Poisson distribution

There were significantly more infections starting in January, March, October, November, and December compared with May and July (*p* < 0.05). The LOESS regression revealed a smooth parabolic curve; therefore, a generalized model with a linear and quadratic term for calendar month was used to determine statistical significance. Both terms were significant (*p* = 0.0014 and *p* = 0.0004, respectively), indicating a significant drop in the frequency of infections during the middle of the calendar year (Fig. 3). Only one of seven SBIs started in a summer month. The number of days hospitalized due to infection was 238 days in the combined studies, with an annualized rate of 0.95 (upper 99% CI limit 1.10) days per patient (Table S3). The annualized rate of days hospitalized due to infection in individual studies ranged from 0.00 to 3.48 (Fig. 2).

In total across all studies, there were 1292 days out of work/school rendering annualized rates of 2.06–8.00 events per patient across individual studies (Table S3).

Overall, there were 9226 days of antibiotic treatment used for infection prophylaxis in all seven studies combined, with an annualized rate of 36.78 (upper 99% CI limit 37.68) days per patient (Table S3). Antibiotic use was much higher in the Japanese study compared with the European and US studies (Fig. 2).

In the Japanese, European, and US pivotal studies, switching to weekly Hizentra® SCIG resulted in an increase in serum IgG trough levels. Patients who received IVIG prior to Hizentra® (including 19 patients in the European pivotal study who received SCIG therapy other than Hizentra®) had median (range) baseline serum IgG trough levels of 5.90 (4.67–10.01), 6.48 (5.26–11.71), and 10.47 (6.54–19.0) g/L, respectively. Median (range) serum IgG trough levels at follow-up studies were 6.64 (5.20–10.43), 8.09 (5.2–11.2), and 11.43 (7.24–22.04) g/L in the Japanese, European, and US pivotal studies, respectively with dose adjustment in the US study (Table 3). Those who switched to Hizentra® SCIG from a previous SCIG had median (range) IgG trough levels in the European pivotal study of 8.73 (5.22–10.15) g/L on Hizentra® vs 8.57 (5.36–10.30) on previous SCIG.

**Table 3:** Serum IgG concentrations

Study	Japanese pivotal (PPS)	Japanese follow-up (PPS)	Japanese extension (PPS)	European pivotal (ITT)	European extension (AT)	US pivotal (MITT)	US extension (ITT)
Total number of patients	21	19	17	46	40	38	21
Baseline serum IgG trough levels, g/L							
Mean (SD)	6.48 (1.46)^a^	7.59 (1.34)^b^	7.89 (1.32)^b^	Previously treated IVIG: 6.78 (1.33)^c, d^ Previously treated SCIG: 8.43 (1.38) ^c, e^ All: 7.49 (1.57)^c^	8.20 (1.32)^b^	10.88 (3.10)^f^	12.20 (3.67)^g^
Median (range)	5.90 (4.67–10.01)^a^	7.39 (5.77–10.61)^b^	8.14 (5.90–9.75)^b^	Previously treated IVIG: 6.48 (5.26–11.71)^d^ Previously treated SCIG: 8.57 (5.36–10.30)^e^ All: 7.02 (5.26–11.71)^c^	8.39 (5.3–11.1)^b^	10.47 (6.54–19.0)^f^	11.30 (7.78–21.01)^g^
Study serum IgG trough levels, g/L							
Mean (SD)	7.15 (1.51)	7.94 (1.54)	8.21 (1.52)	8.10 (1.34)	7.97 (1.17)	12.53 (3.21)	11.98 (3.65)
Median (range)	6.64 (5.20–10.43)	7.64 (6.02–11.70)	8.13 (5.64–11.08)	Previously treated IVIG: 7.72 (5.87–11.15) Previously treated SCIG: 8.73 (5.22–10.15) All: 8.09 (5.2–11.2)	8.12 (5.8–11.1)	11.43 (7.24–22.04)	11.19 (6.88–22.11)

*AT* all treated, *IgG* immunoglobulin G, *ITT* intention-to-treat, *IVIG* intravenous immunoglobulin, *MITT* modified intention-to-treat, *PPS* per protocol set, *SCIG* subcutaneous immunoglobulin, *SD* standard deviation

^a^During the last 3 months prior to study enrollment

^b^Week 1 of the follow-up/extension study

^c^Mean of individual median pre-study serum IgG trough values, based on the last 3 IgG trough values (or less if 3 values were not available) before the first Hizentra® infusion

^d^*n* = 27

^e^*n* = 19

^f^Screening visit

^g^IgG levels up to 90 days prior to screening visit were taken into account

In the European pivotal study, the primary objective of sustained serum IgG levels with Hizentra® similar to the patients’ previous IgG treatment was clearly met. The mean of individual median IgG trough values with Hizentra® treatment was slightly higher in patients with CVID (8.37 g/L during infusions 12 to 17) than in patients with XLA (7.61 g/L during infusions 12 to 17); however, the increase was comparable (6.9% in CVID compared to 8.7% in XLA). The primary objective of the Japanese pivotal study was also met, as the GMR of serum IgG trough levels was similar to those of the preceding IVIG treatment period (GMR = 1.09; 90% CI 1.06–1.14). All three pivotal studies, therefore, met their respective primary objectives.

There were no clinically relevant differences between median serum IgG trough concentrations at baseline and during SCIG maintenance dosing in the follow-up and extension studies (Table 3).

### Pharmacokinetics

The final structural population PK (pharmacometric) model was a two-compartment model with inter-individual variability on CL and V2 on all patients. The median values of all non-covariate parameters from bootstrap resampling were consistent with the original population PK estimates (Table S4). The effect of body weight on CL and V2 was within the 90% CI but was more variable, and the CIs for these parameters were relatively wide (Table S4).

Analysis of pooled data revealed no differences in IgG metabolism between ethnic groups despite the numerically different results. The effect of the Japanese population was also tested in various sensitivity models, results from which showed that there was no significant race-related covariate effect from the inclusion of Japanese patients (Fig. S2). After adjustment for average body weight, major PK variables affecting IgG half-life, such as CL and V2, were the same in the Caucasian and Asian patient populations (unpublished data).

### Safety

There were 5039 AEs in total across all studies. Overall, there were no relevant differences in the frequency of AEs between the different age groups receiving Hizentra® in any of the studies, and there was no increase in the rate of AEs with increasing age, as might be expected. The rates of AEs per infusion were also similar in male and female patients. The incidence of patients with AEs at least possibly related to the study medication in the European pivotal study appeared higher in patients with CVID compared with patients with XLA (23 [76.7%] vs. 8 [40.0%], respectively).

**Fig. 4:**
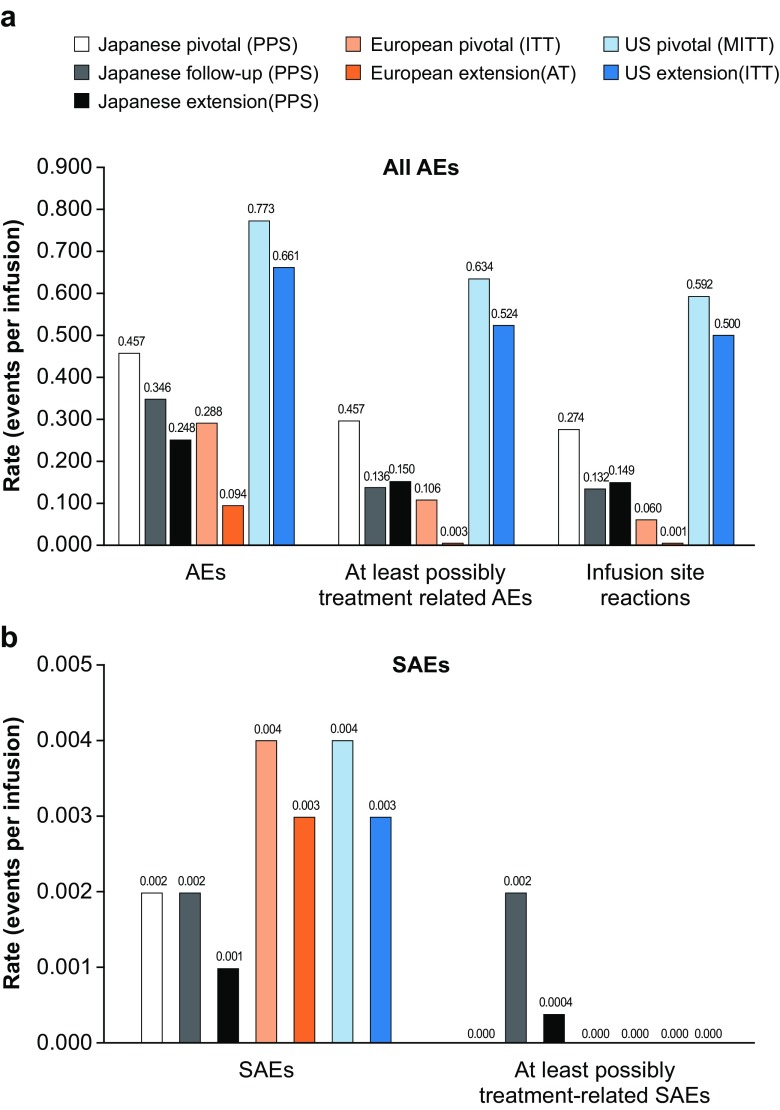
Summary of AEs (**a**) and SAEs (**b**). AE adverse event, AT all treated, ITT intention-to-treat, SAE serious adverse event

Events per infusion ranged from 0.094 in the European extension study to 0.773 in the US pivotal study (Fig. 4a). While most patients experienced ≥1 AE, most were mild/moderate. There was a single reaction that required infusion interruption during treatment.

In total, 3197 treatment-related AEs were reported (0.003–0.634 AEs per infusion in the combined studies; Table S5). As expected for subcutaneous administration, the most common treatment-related AEs were ISRs (2919 events; 0.001–0.592 AEs per infusion in the combined studies; Table S5). The rate of ISRs was variable amongst the studies, a phenomenon that may reflect the differences in assessment.

Systemic AEs were uncommon. Of 45 serious AEs (SAEs) reported (0.001–0.004 SAEs per infusion in the combined studies; Fig. 4b), two were considered possibly related to the study medication (one case of encephalitis in the Japanese follow-up study and one case of asthma in the Japanese extension study); however, viral infection was considered a plausible alternative explanation for the episode of encephalitis. In the European extension study, one patient with a known ongoing history of recurrent severe pneumonia developed an acute exacerbation and subsequently died of respiratory failure. This death was thought to be related to underlying disease and not to the study medication.

The incidence of AEs leading to discontinuation was low in all studies. No patients in the Japanese pivotal study and one patient each in the Japanese follow-up study (encephalitis) and Japanese extension study (local ISRs) discontinued the study due to AEs. In the European studies, 6 patients (11.8%) in the pivotal study discontinued due to AEs (at least possibly related to study drug in 3 patients) and 1 patient died (due to AE unrelated to study drug) in the extension study. In the US studies, 2 patients (4.1%) in the pivotal study discontinued due to AEs (one of which was deemed at least possibly related to study drug), and 1 patient (4.8%) in the US extension study discontinued due to an AE, which was not considered related to study drug.

### Comparison of Privigen® and Hizentra®

ISRs were more common with Hizentra® than with Privigen® (ratio 90.8; 95% CI 35.27–233.51; Table S6), although only occurring with a rate of approximately 0.2 per infusion. However, systemic AEs such as fatigue, headache, vomiting, nausea, and pyrexia were more common with Privigen® (≤0.22 events/infusion) than with Hizentra® (≤0.00915 events/infusion). Ratios were ≤0.0981, indicating an approximately 10-fold lower incidence of these events in patients receiving Hizentra®. These results underline the expected outcome that ISRs are more common with SCIG than IVIG, while systemic reactions are, conversely, less common with SCIG than with IVIG.

## Discussion

This review of seven Phase 3 clinical trials in 125 patients who received 15,013 weekly infusions for a total observation period of 250.9 patient years supports that SCIG Hizentra® administration as a treatment is effective against infections, particularly SBIs, in a broad age range of patients with PID.

Weekly SCIG Hizentra® administration increased serum IgG trough levels compared with equivalent monthly doses of IVIG. Maintenance serum IgG trough levels were similar to those associated with previous SCIG therapy. These results are in agreement with the known advantages of SCIG compared to IVIG and confirm the well-recognized increase in trough IgG following a dose-equivalent switch from IVIG to SCIG [1, 5]. In addition, a previous pharmacometric modeling and simulation study, which partly used data from the US and European pivotal trials, showed that weekly or biweekly SCIG dosing would produce serum IgG levels within 10% of those achieved with 4-weekly IVIG dosing [19]. Furthermore, pharmacometric analysis showed that a range of subcutaneous dosing schedules (from daily to biweekly) for the same total equivalent weekly dose that provide similar serum IgG levels [22] can be implemented.

Annualized infection rates, SBIs, and days spent in hospital due to infection were low, and compared favorably to findings of previous studies that examined the effects of other SCIG therapies in PID [23]. The annualized SBI rates captured in the combined European and US extension studies were well below the accepted US FDA and European Medicines Agency threshold of 1 SBI per patient year [14]. No patients were reported to have SBIs in the US pivotal study, EU pivotal study, or any of the Japanese studies. The highest annualized rate of SBIs was only 0.06 infections per patient per year (observed in the US extension study) which is lower than the previously-reported annualized rate of 0.08 with IVIG for the treatment of PID [24]. Since patients who receive adequate IgG replacement therapy may still experience occasional serious infections, especially in those with pre-existing conditions such as bronchiectasis, it is expected that some SBIs will be captured within the time frame of these extension studies, and formed part of the purpose of analysis of this large dataset [25, 26].

The majority of patients, including those who experienced a SBI, had serum IgG trough levels within the normal range. Also, clear seasonal patterns of infection frequency and severity were observed in these patients. This suggests that increased monitoring during the months from October to February, which are traditionally associated with higher infection risk, may be advisable for patients with PID. Further research is needed to evaluate if seasonally-optimized SCIG doses and/or targeted use of prophylactic antibiotics during this period would be beneficial. It may also be that IgG replacement therapy does not offer patient as much protection from upper airway viral infections as it does protection from pneumonia [27].

The rates of hospitalization were higher in the European studies compared with the Japanese and US studies. However, results from the European studies were disproportionally affected by the experience of a single 5-year-old female patient who experienced three SAEs that resulted in 71 school days missed and 63 days spent in hospital [13]. Excluding this patient, the number of days hospitalized decreased from 3.48 to 0.95 days per patient per year [13]. Another possible factor concerning the interpretation of these findings is that the threshold for hospitalization and duration of in-hospital stay may be lower in many European countries compared with the US and other countries as a function of differing practice standards.

SCIG has previously been shown to be associated with a lower rate of AEs than IVIG. Several open-label prospective studies in the US and Europe have reported a zero incidence of SAEs related to SCIG [28–30]. In previously-published studies, the incidence of SAEs related to IVIG was reported to range 9–29% [31–33]. The present analysis showed a similar incidence of SAEs, ranging from 4% in Japanese pivotal study to 35% in European extension study. SAEs reported were unrelated to study drug across all studies with the exception of 1 SAE each in the Japanese follow-up study (encephalitis) and extension study (asthma). However, it is difficult to draw meaningful conclusions due to potential differences in the measurement and recording of AEs, patient demographic characteristics, and treatment parameters.

In line with previous studies, Hizentra® was well tolerated. AEs were predominately mild or moderate, and mostly ISRs. The safety profile of Hizentra® was similar to that of other subcutaneous IgG replacement therapies, in terms of the type, frequency, and treatment-relatedness of AEs [34, 35]. The comparison of AEs observed in patients receiving IVIG and SCIG included in this analysis confirms the expected outcome that ISRs are more common, while systemic reactions are less common, with SCIG than IVIG.

Interestingly, the ISR rates differed widely across the seven trials, as did the assessment methods, time points, and scales for reporting ISRs. The rate of ISRs in the US studies [12, 14] appeared higher in comparison with the Japanese and European studies. In this regard, the US pivotal study was the first clinical trial using Hizentra®, and ISRs were comprehensively evaluated by both the patient and the investigator, using a variety of methods at multiple mandatory time points and with leading questions during and after infusion [12]. Most ISRs were mild and transient, did not require treatment, and were otherwise felt to be expected consequences of simply infusing fluid into the subcutaneous tissue. These findings lead to adjustments to less stringent evaluation criteria in subsequent studies with Hizentra® and other SCIGs [13–15, 34, 35]. The reported incidences and rates of infusion site AEs in the European and Japanese studies were also similar to those reported in studies using an alternative, recently-licensed, 20% IgG therapy, including those reported in US patients [36, 37]. In the European pivotal study, the rate of temporally-associated AEs was similar for all starting infusion rates (0.142 in the <15 mL/h group, 0.172 in the 15–25 mL/h group, and 0.191 in the >25 mL/h group). Across US studies, no trends in overall AE rates per infusion rate were reported, with the exception of a lower AE rate of 0.686 in the group with the highest infusion rates of >25 mL/h, compared with a higher AE rate of 0.887 with the lower infusion rates of 15–25 mL/h in the US pivotal study, although these differences were not compared statistically. In the Japanese pivotal study, the overall AE rate was 0.362 for patients receiving SCIG at a rate of 15–25 mL/h and 0.286 for those at >25 mL/h. In the Japanese follow-up study, the rate of all temporally-associated AEs for infusions of 15–25 mL/h was 0.150, compared with 0.298 for >25 mL/h, however the limited number of patients reporting a high number of AEs in either infusion rate group and the uneven distribution of AEs means that these results should be interpreted with caution. No trends were observed in the Japanese extension study. The lack of comparative, head-to-head trials between available 20% IgG replacement products requires thoughtful consideration of differences in reporting methodology that may contribute significantly to reported differences in adverse reactions rates. These findings also emphasize that standardization of definitions and timing of infusion site AE capture amongst in different countries and different companies could be beneficial for future studies.

The stability of serum IgG concentrations during SCIG maintenance phases (i.e., during the Japanese follow-up study and the Japanese, European, and US extension studies) suggests that adherence to effective dosing and delivery was excellent in these cohorts. Furthermore, the low numbers of patients discontinuing treatment suggested that Hizentra® SCIG was also well tolerated.

There were some differences between the Japanese, European, and US studies. These include distribution of primary endpoints, study designs (mandatory IVIG treatment period in the Japanese pivotal study only), study length, demographics, and SCIG dose (calculated using a conversion factor of 1.53 in the US pivotal study vs an equivalent dose to previous IVIG therapy in the European and Japanese pivotal studies). Furthermore, the use of prophylactic antibiotics appeared higher in the Japanese studies, most likely as a result of local prescribing practices.

## Conclusions

Long-term tolerability of IgG replacement therapy in PID is an important consideration, as patients often require IgG throughout life. The results of these studies indicate that repeated, self-administered SCIG Hizentra® therapy up to 4 years is efficacious and well tolerated.

## Electronic Supplementary Material

ESM 1 (DOCX 348 kb)
