# Supplementary material for: Long-Term Efficacy and Safety of Hizentra® in Patients with Primary Immunodeficiency in Japan, Europe, and the United States: a Review of 7 Phase 3 Trials
Source: J Clin Immunol. 2018 Nov 10;38(8):864–75. doi: 10.1007/s10875-018-0560-5 (PMC6292970; doi:10.1007/s10875-018-0560-5)

# Supplementary Material

# Title: Long-term Efficacy and Safety of Hizentra® in Patients with Primary Immunodeficiency in Japan, Europe, and the United States: a Review of 7 Phase 3 Trials

Stephen R. Jolles^1^, Mikhail A. Rojavin^2^, John-Philip Lawo^3^, Robert Nelson Jr^4^, Richard L. Wasserman^5^, Michael Borte^6^, Michael A. Tortorici^2^, Kohsuke Imai^7^, Hirokazu Kanegane^8^

^1^University Hospital of Wales, Cardiff, UK; ^2^CSL Behring LLC, King of Prussia, PA, USA; ^3^CSL Behring GmbH, Marburg, Germany; ^4^Indiana University School of Medicine and the Melvin and Bren Simon Cancer Center, Indianapolis, IN, USA; ^5^Medical City Children’s Hospital, Dallas, TX, USA; ^6^Hospital St. Georg GmbH Leipzig, Academic Teaching Hospital of the University of Leipzig, Leipzig, Germany; ^7^Department of Community Pediatrics, Perinatal and Maternal Medicine, Tokyo Medical and Dental University (TMDU), Tokyo, Japan; ^8^Department of Pediatrics and Developmental Biology, Graduate School of Medical and Dental Sciences, Tokyo Medical and Dental University (TMDU), Tokyo, Japan

| Corresponding author | Stephen Jolles |
| --- | --- |
| Corresponding address | Immunodeficiency Centre for Wales, University Hospital of Wales, Cardiff, UK |

Journal: *Journal of Clinical Immunology*

## Japanese follow-up and extension studies: methods

### Study design

The Japanese follow-up study was a prospective multicenter open-label, single-arm phase 3 study following on from the Japanese pivotal study. Patients were then enrolled into the Japanese extension study, a prospective multicenter, open-label, post-marketing approval study. Both studies investigated the efficacy and safety of Hizentra® in patients with primary immunodeficiency (PID).

### Patient population

Patients were eligible if they had participated in the preceding study (Japanese pivotal study for the follow-up study and follow-up study for the extension study) and had tolerated Hizentra® treatment well. Patients were excluded from either study if they met any of the following exclusion criteria: (1) female patients of childbearing potential either not using, or not willing to use, a medically reliable method of contraception for the entire duration of the study, or not sexually abstinent for the entire duration of the study, or not surgically sterile; (2) intention to become pregnant during the course of the study; (3) pregnancy or nursing mother; (4) participation in a study with an investigational medicinal product within 3 months prior to enrollment except for the preceding Hizentra® study; (5) patients planning to donate blood during the study; (6) study personnel who were involved in the development of Hizentra®, or employees at the study site; and subjects who were relatives or spouses of the study personnel; (7) any condition that was likely to interfere with evaluation of Hizentra® or satisfactory conduct of the study; (8) known or suspected antibodies to Hizentra® or its excipients.

Addition exclusion criteria for entering the follow-up study included: (1) ongoing serious bacterial infection (SBIs) (pneumonia, bacteremia/septicemia, osteomyelitis/septic arthritis, bacterial meningitis, or visceral abscess) at the time of the first infusion; (2) hypoalbuminemia, protein-losing, enteropathies, and any proteinuria (known total urine protein concentration >0.2 g/L or urine protein ++ by dipstick); (3) re-entry of subjects who previously discontinued treatment during the follow-up study.

### Study drug administration

Patients received home-based subcutaneous Hizentra® (with the exception of mandatory supervised infusions at the study site during the follow-up study at Weeks 1, 12, and 24, and optional supervised infusions at Weeks 4, 8, 16, and 20 during the follow-up study) at a dose equivalent to the patient’s last dose in the preceding study as recommended by the investigator. In the follow-up study, doses could be adjusted if medically indicated, or to result in immunoglobulin G (IgG) trough levels of ≥5 g/L. In the extension study, doses could be adjusted if medically indicated or when patient weight changed >±5%. Infusions were given weekly in both studies (up to 24 weekly doses in the follow-up study and up to 36 months of weekly doses in the extension study).

### Study schedule

In the follow-up study patients underwent clinical and laboratory assessments at Weeks 1, 4, 8, 12, 16, 20, and 24. Mandatory site visits occurred on Weeks 1, 12, and 24. In the extension study patients underwent clinical and laboratory assessments during clinic visits at Weeks 1, 12, 24, 36, 48, 60, 120, 132, and 144. During both studies, adverse events (AEs) were recorded during clinic visits, and throughout the study period at home in a patient diary.

### Study objectives and endpoints

Both studies investigated the long-term efficacy and safety of Hizentra®. Efficacy endpoints in both studies were serum IgG trough concentrations; the number of infection episodes (primary efficacy endpoint of the extension study; the follow-up study did not have a primary efficacy endpoint) the number and annualized rate of SBIs; the number, rate, and annualized rate of infections; days of hospitalization or out of work/school/kindergarten/day care due to infection and the use of antibiotics for infection prophylaxis and treatment.

In the follow-up study the primary safety endpoint was the individual rates of AEs per infusion and secondary safety endpoints were the overall rate of AEs per infusion, the incidence of AEs per patient and local tolerability of each infusion. In the extension study safety endpoints were the incidence of AEs per subject and the overall rate of AEs per infusion.

All efficacy analyses were performed on the per protocol sets (PPSs), which included all patients who received uniformly repeated weekly Hizentra® infusions and had ≥1 documented total serum IgG trough level during the study in question. All safety analyses were performed on the all-treated (AT) dataset, which included all patients who had received at least 1 Hizentra® dose during the study in question.

# Supplementary Tables

## Table S1 Study objectives and endpoints

| **Study** | **Japan pivotal** | **Japan follow-up** | **Japan extension** | **European pivotal** | **European extension** | **US pivotal** | **US extension** |
| --- | --- | --- | --- | --- | --- | --- | --- |
| **Primary objective^a^** | Evaluate if sustained total serum IgG trough levels with SCIG Hizentra® similar to preceding IVIG treatment period in the PPS could  be attained | Evaluate the rate, severity, and relatedness of newly developing or worsening AEs per infusion during the treatment period | Evaluate the number  of infection episodes  (serious and non-serious) during the study | Achieve sustained total serum IgG trough levels similar to the previous IgG treatment in the ITT population | Continue assessing the efficacy, tolerability, safety, and, additionally, long-term HRQoL of Hizentra® in patients with PID, who elected to continue the treatment they received previously in the European pivotal study | Evaluate whether the annual rate of SBIs per subject was <1 | Assess the efficacy, tolerability, and safety of Hizentra® in patients with PID who elected to continue treatment with Hizentra® previously received in the US pivotal study |
| **Secondary efficacy objectives** | - To assess:   - Number of infection episodes (serious and non-serious)   - Number of days out of work/school   - Number of days of hospitalization due to infections   - Duration of antibiotic use for infection prophylaxis and treatment | - Maintenance of consistent IgG serum levels - To assess:   - Rate of clinically documented SBIs   - Number of infection episodes   - Number of days out of work/school   - Number of days of hospitalization due to infections   - Duration of antibiotic use for infection prophylaxis and treatment | - To assess:   - Annualized rate of clinically documented SBIs   - Number of days out of work/school   - Number of days of hospitalization due to infections   - Duration of antibiotic use for infection prophylaxis and treatment   - Serum IgG concentrations | - To assess:   - Rate of clinically documented SBIs   - Number of infection episodes   - Number of days out of work/school   - Number of days of hospitalization due to infections   - Duration of antibiotic use for infection prophylaxis and treatment | - Maintenance of total serum IgG trough levels consistent with the European pivotal - To assess:   - Rate of clinically documented SBIs   - Number of infection episodes   - Number of days out of work/school   - Number of days of hospitalization due to infections   - Duration of antibiotic use for infection prophylaxis and treatment | - To assess:   - Annualized rate of SBIs     - Number of infection episodes (serious and non-serious)   - Number of days out of work/school   - Number of days of hospitalization due to infections   - Duration of antibiotic use for infection prophylaxis and treatment   - Total serum IgG trough levels | - To assess:   - Annualized rate of SBIs   - Total serum IgG trough levels   - Number of infection episodes   - Number of days out of work/school   - Number of days of hospitalization due to infections   - Duration of antibiotic use for infection prophylaxis and treatment |
| **Secondary safety objectives** | - To assess:   - Number, rate, severity, and relatedness of any AEs per infusion and subject   - Local tolerability of SCIG infusions   - Vital sign changes before and after infusions at the study site   - Changes in routine laboratory parameters (blood chemistry, hematology, urinalysis) compared with baseline assessments   - Changes in viral safety markers of Hizentra® treatment as compared with baseline assessments | - To assess:   - Overall rate of AEs per infusion, assessed in total, by severity and by causality relationship to study medication   - Incidence of AEs per subject, assessed in total, by severity and by causality relationship to study medication   - Local tolerability of each infusion, as assessed by the subject 24–72 hours after the infusion, using the categories | - To assess:   - Incidence of AEs per subject, assessed in total, by severity and by causal relationship to study medication   - Overall rate of AEs per infusion, assessed in total, by severity and by causal relationship to study medication | - To assess:   - Local tolerability of SC infusions   - Overall rate of AEs per infusion, assessed in total, by severity and by causality relationship to study medication   - Vital sign changes before and after infusions at the study site   - Changes in routine laboratory parameters as compared with baseline assessments | - To assess:   - Rate, severity, and relatedness of any AEs per infusion and subject   - Changes in vital signs, as compared to baseline assessments   - Changes in routine laboratory parameters as compared with baseline assessments | - To assess:   - Rate, intensity, and relatedness of any AEs per subject and infusion     - Local tolerability in terms of injection site reactions   - Changes in clinical laboratory parameters as compared with baseline assessments     - Changes in physical examination results as compared with screening assessments     - Vital sign changes     - Changes in concomitant medications   - Changes in viral safety markers as compared with baseline assessments | - To assess:   - Local tolerability in terms of injection site reactions   - Rate, intensity, and relatedness of any AEs   - Changes in clinical laboratory parameters as compared with baseline assessments   - Changes in physical examination results as compared with screening assessments   - Vital sign changes   - Changes in concomitant medications   - Changes in viral safety markers as compared with baseline assessments |

AEs, adverse events; HRQoL, health-related quality of life; IgG, immunoglobulin G; ITT, intention-to-treat; IVIG, intravenous immunoglobulin; PID, primary immunodeficiency; PPS, per protocol set; SBIs, serious bacterial infections; SC, subcutaneous; SCIG, subcutaneous immunoglobulin.
^a^In the European and US extension studies, there was no primary objective; instead, the “overall study objective” is reported here

## Table S2 Analysis data sets

| **Study** | **Population used for efficacy analyses** | | **Population used for safety analyses** | | |
| --- | --- | --- | --- | --- | --- |
|  | **Name** | **Definition** | | **Name** | **Definition** |
| **Japan pivotal** | PPS | All patients who had received ≥6 doses of IVIG at 3–4 weekly intervals^a^ followed by weekly Hizentra® treatments until at least Week 16, with ≥1 documented total serum IgG trough level recorded during the efficacy period | | AT | All patients treated with ≥1 dose of IVIG or Hizentra® during the study |
| **Japan follow-up** | PPS | All patients who received uniform weekly Hizentra® infusions and had ≥1 documented total serum IgG trough level during the study | | AT | All patients treated with Hizentra®  during the study |
| **Japan extension** | PPS | All patients who received uniform weekly Hizentra® infusions and ≥1 documented total serum IgG trough level during the study | | AT | All patients treated with Hizentra®  during the study |
| **European pivotal** | ITT | All patients who received Hizentra®  during the efficacy period | | AT | All patients treated with Hizentra®  during the study |
| **European extension** | AT | All patients who received Hizentra®  during the efficacy period | | AT | All patients treated with Hizentra®  during the study |
| **US pivotal** | MITT | All patients who received Hizentra®  during the efficacy period | | ITT | All patients treated with Hizentra®  during the study |
| **US extension** | ITT | All patients who received Hizentra®  during the study | | AT | All patients treated with Hizentra®  during the study |

AT, all-treated; IgG, immunoglobulin G; IVIG, intravenous immunoglobulin; ITT, intention-to-treat; MITT, modified intention-to-treat; PPS, per-protocol set.
^a^Pre-study and during the IVIG study period

## Table S3 Infections and days hospitalized or out of work/school/kindergarten/day care due to infection

| **Study** | **Japan pivotal (PPS)** | **Japan follow-up  (PPS)** | **Japan extension  (PPS)** | **European pivotal (ITT)** | **European extension (AT)** | **US pivotal (MITT)** | **US extension  (ITT)** | **Overall** |
| --- | --- | --- | --- | --- | --- | --- | --- | --- |
| **Total number of patients** | 21 | 19 | 17 | 46 | 40 | 38 | 21 |  |
| **Total number of study days** | 1,840 | 3,214 | 14,913 | 8,745 | 38,208 | 12,697 | 11,950 | 91,567 |
| **SBIs** |  |  |  |  |  |  |  |  |
| Number of patients (%) | 0 (0.0) | 0 (0.0) | 0 (0.0) | 0 (0.0) | 5 (12.5) | 0 (0.0) | 2 (9.5) |  |
| Number of events (annualized rate, upper 1-sided 99% CI) | 0 (0.00, 0.914) | 0 (0.00, 0.523) | 0 (0.00, 0.113) | 0 (0.00, 0.192) | 5 (0.05, 0.125) | 0 (0.00, 0132) | 2 (0.06, 0.257) | 7 (0.03, 0.064) |
| **All infections** |  |  |  |  |  |  |  |  |
| Number of patients (%) | 11 (52.4) | 14 (73.7) | 16 (94.1) | 36 (78.3) | 38 (95.0) | 31 (81.6) | 20 (95.2) |  |
| Number of events (annualized rate, upper 1-sided 99% CI) | 15 (2.98, 5.305) | 38 (4.32, 6.244) | 78 (1.91, 2.475) | 124 (5.18, 6.364) | 349 (3.33, 3.773) | 96 (2.76, 3.489) | 78 (2.38, 3.089) | 778 (3.10, 3.370) |
| **Days hospitalized due to infection^a^** |  |  |  |  |  |  |  |  |
| Number of patients (%) | 1 (4.8) | 0 (0.0) | 2 (11.8) | 4 (8.7)^b^ | 7 (17.5) | 1 (2.6) | 2 (9.5) |  |
| Number of days (annualized rate, upper 1-sided 99% CI) | 3 (0.55, 1.842) | 0 (0.00, 0.523) | 14 (0.34, 0.623) | 86 (3.48, 4.451)^b^ | 110 (1.06, 1.314) | 7 (0.2, 0.463) | 18 (0.55, 0.934) | 238 (0.95, 1.100) |
| **Days out of work/school^c^** |  |  |  |  |  |  |  |  |
| Number of patients (%) | 7 (33.3) | 7 (36.8) | 13 (76.5) | 20 (43.5)^b^ | 27 (67.5) | 12 (31.6) | 9 (42.9) |  |
| Number of days (annualized rate, upper 1-sided 99% CI) | 19 (3.48, 5.841) | 25 (2.84, 4.464) | 133 (3.26, 3.975) | 198 (8.00, 9.426)^b^ | 706 (6.77, 7.390) | 71 (2.06, 2.699) | 140 (4.28, 5.195) | 1292 (5.14, 5.482) |
| **Prophylactic antibiotic use** |  |  |  |  |  |  |  |  |
| Number of patients, n (%) | 5 (23.8) | 6 (31.6) | 6 (35.3) | 4 (8.7) | 6 (15.0) | 2 (5.3) | 6 (28.6) |  |
| Number of days, n (annualized rate, upper 1-sided 99% CI) | 422 (83.71, 93.692) | 991 (112.54, 121.145) | 4,490 (109.89, 113.770) | 297 (12.40, 14.175) | 2,021 (19.31, 20.329) | 16 (0.46, 0.806) | 989 (30.21, 32.519) | 9,226 (36.78, 37.677) |

AT, all-treated; CI, confidence interval; ITT, intention-to-treat; MITT, modified intention-to-treat; PPS, per protocol set; SAE, serious adverse event; SBI, serious bacterial infection.^a^For all studies except the US extension study, the number of days from patient diaries was different to the number of study days: 1,990 for the Japanese pivotal, 3,214 for the Japanese follow-up study, 9,033 for the European pivotal study, 38,045 for the European extension study, and 12,605 for the US pivotal study. ^b^These results are significantly affected by input from a single patient, a 5-year old girl who experienced 3 SAEs resulting in 71 days missed from school and 63 days spent in hospital. ^c^Days out of work/school/kindergarten/day care or unable to perform normal activities due to infections

## Table S4 Point estimates and 90% confidence intervals for the final population pharmacokinetic parameter estimates from bootstrap resampling

| **Parameter** | **Parameter description** | **Point population estimate** | **Bootstrap resampling final population pharmacokinetic model median (5^th^, 95^th^ percentiles)** |
| --- | --- | --- | --- |
| θ1 | Bioavailability fraction, F1 | 0.676 | 0.676 (0.621, 0.733) |
| θ2 | Clearance, CL (L/d) | 0.141 | 0.141 (0.131, 0.15) |
| θ3 | Volume of distribution in central compartment, V2 (L) | 4.19 | 4.15 (3.57, 4.72) |
| θ4 | Apparent volume of distribution in peripheral compartment, V3 (L) | 2.63 | 2.67 (1.56, 3.54) |
| θ5 | Apparent inter-compartmental clearance, Q (L/d) | 0.114 | 0.113 (0.0773, 0.194) |
| θ6 | Absorption rate constant, KA (d-1) | 0.393 | 0.396 (0.332, 0.456) |
| θ7 | Effect of body weight on CL | 0.799 | 0.874 (0.704, 0.989) |
| θ8 | Effect of body weight on V2 | 0.477 | 0.615 (0.332, 0.942) |
| θ9 | Baseline endogenous IgG level in treatment naïve subjects (Study ZLB06_005) (g/L) | 4.10 | 4.09 (3.57, 4.59) |

CL, clearance of human immunoglobulin; F1, subcutaneous bioavailability; KA, absorption rate constant; Q, inter-compartmental clearance; V2, volume of distribution of the central compartment; V3, volume of distribution of the peripheral compartment; θ, population (fixed effect) parameter

## Table S5 Adverse events

| **Study** | **Japan pivotal  (AT)** | **Japan follow-up  (AT)** | **Japan extension  (AT)** | **European pivotal  (AT)** | **European extension  (AT)** | **US pivotal  (ITT)** | **US extension  (AT)** |
| --- | --- | --- | --- | --- | --- | --- | --- |
| **Total number of patients** | 25 | 23 | 22 | 51 | 40 | 49 | 21 |
| **Total number of infusions** | 584 | 529 | 2,665 | 1,831 | 5,405 | 2,264 | 1,735 |
| **AEs overall** |  |  |  |  |  |  |  |
| Number of patients (%) | 24 (96.0) | 22 (95.7) | 22 (100.0) | 50 (98.0) | 39 (97.5) | 49 (100.0) | 21 (100.0) |
| Number of events (rate per infusion) | 267 (0.457) | 183 (0.346) | 660 (0.248) | 527 (0.288) | 506 (0.094) | 1,749 (0.773) | 1,147 (0.661) |
| **At least possibly treatment-related AEs** |  |  |  |  |  |  |  |
| Number of patients (%) | 21 (84.0) | 10 (43.5) | 13 (59.1) | 31 (60.8) | 8 (20.0) | 49 (100.0) | 21 (100.0) |
| Number of events (rate per infusion) | 173 (0.296) | 72 (0.136) | 399 (0.150) | 194 (0.106) | 14 (0.003) | 1,436 (0.634) | 909 (0.524) |
| **Injection site reactions** |  |  |  |  |  |  |  |
| Assessment | Patient/caregiver 24–72 h after infusion | Patient/caregiver 24–72 h after infusion | Patient/caregiver time point not specified | Patient/caregiver 24–72 h after infusion | Patient/caregiver time point not specified | Investigator 15–45 mins;  Patient/caregiver 24±3 h after infusion | Patient/caregiver 24±3 h after infusion |
| Number of patients (%) | 20 (80.0) | 8 (34.8) | 12 (54.5) | 25 (49.0) | 6 (15.0) | 49 (100.0) | 19 (90.5) |
| Number of events (rate per infusion) | 160 (0.274) | 70 (0.132) | 398 (0.149) | 110 (0.060) | 7 (0.001) | 1,341 (0.580) | 868 (0.500) |
| **SAEs** |  |  |  |  |  |  |  |
| Number of patients (%) | 1 (4.0) | 1 (4.3) | 2 (9.1) | 5 (9.8) | 14 (35.0) | 7 (14.3) | 4 (19.0) |
| Number of events (rate per infusion) | 1 (0.002) | 1 (0.002) | 3 (0.001) | 7 (0.004) | 18 (0.003) | 10 (0.004) | 5 (0.003) |
| **At least possibly treatment-related SAEs** |  |  |  |  |  |  |  |
| Number of patients (%) | 0 (0.0) | 1 (4.3) | 1 (4.5) | 0 (0.0) | 0 (0.0) | 0 (0.0) | 0 (0.0) |
| Number of events (rate per infusion) | 0 (0.000) | 1 (0.002) | 1 (0.000) | 0 (0.000) | 0 (0.000) | 0 (0.000) | 0 (0.000) |

AE, adverse event; AT, all-treated; ITT, intention-to-treat; SAE, serious adverse event

## Table S6 Adverse events comparison between Hizentra® and Privigen®

| **Term** | **Treatment** | **Number of events** | **Number of infusions** | **Events per infusion** | **Ratio (Hizentra®/Privigen®)** | **95 % CI for ratio** | |
| --- | --- | --- | --- | --- | --- | --- | --- |
|  |  |  |  |  |  | Lower bound | Upper bound |
| Injection/infusion site reactions | Hizentra® | 2949 | 14,696 | 0.20067 | 90.7516 | 35.2705 | 233.506 |
|  | Privigen® | 4 | 1809 | 0.00221 |  |  |  |
| Fatigue | Hizentra® | 47 | 14,696 | 0.00320 | 0.0981 | 0.0670 | 0.144 |
|  | Privigen® | 59 | 1809 | 0.03261 |  |  |  |
| Headache | Hizentra® | 133 | 14,696 | 0.00905 | 0.0410 | 0.0337 | 0.050 |
|  | Privigen® | 399 | 1809 | 0.22056 |  |  |  |
| Nausea | Hizentra® | 15 | 14,696 | 0.00102 | 0.0369 | 0.0209 | 0.065 |
|  | Privigen® | 50 | 1809 | 0.02764 |  |  |  |
| Vomiting | Hizentra® | 12 | 14,696 | 0.00082 | 0.0301 | 0.0162 | 0.056 |
|  | Privigen® | 49 | 1809 | 0.02709 |  |  |  |
| Pyrexia | Hizentra® | 25 | 14,696 | 0.00170 | 0.0488 | 0.0309 | 0.077 |
|  | Privigen® | 63 | 1809 | 0.03483 |  |  |  |

CI, confidence interval

# Figure legends

**Fig. S1** Patient disposition. IVIG, intravenous immunoglobulin, SCIG, subcutaneous immunoglobulin

**Fig. S2** Comparison of simulated steady state IgG concentration profiles in non-Japanese subjects on adjusted Japanese trial dosing and non-Japanese trial dosing. Light gray shade shows confidence intervals of IgG levels with adjusted Japanese trial dosing; black dashed lines show CIs of relevant non-Japanese trials. The median simulated profile of the adjusted Japanese trial dosing and relevant non-Japanese trials are in solid gray and black lines, respectively. Observed IgG levels in non-Japanese trials (black diamonds) are shown for comparison. The black dotted line around 5 g/L shows XX. CI, confidence interval; IgG, immunoglobulin G

## Fig. S1


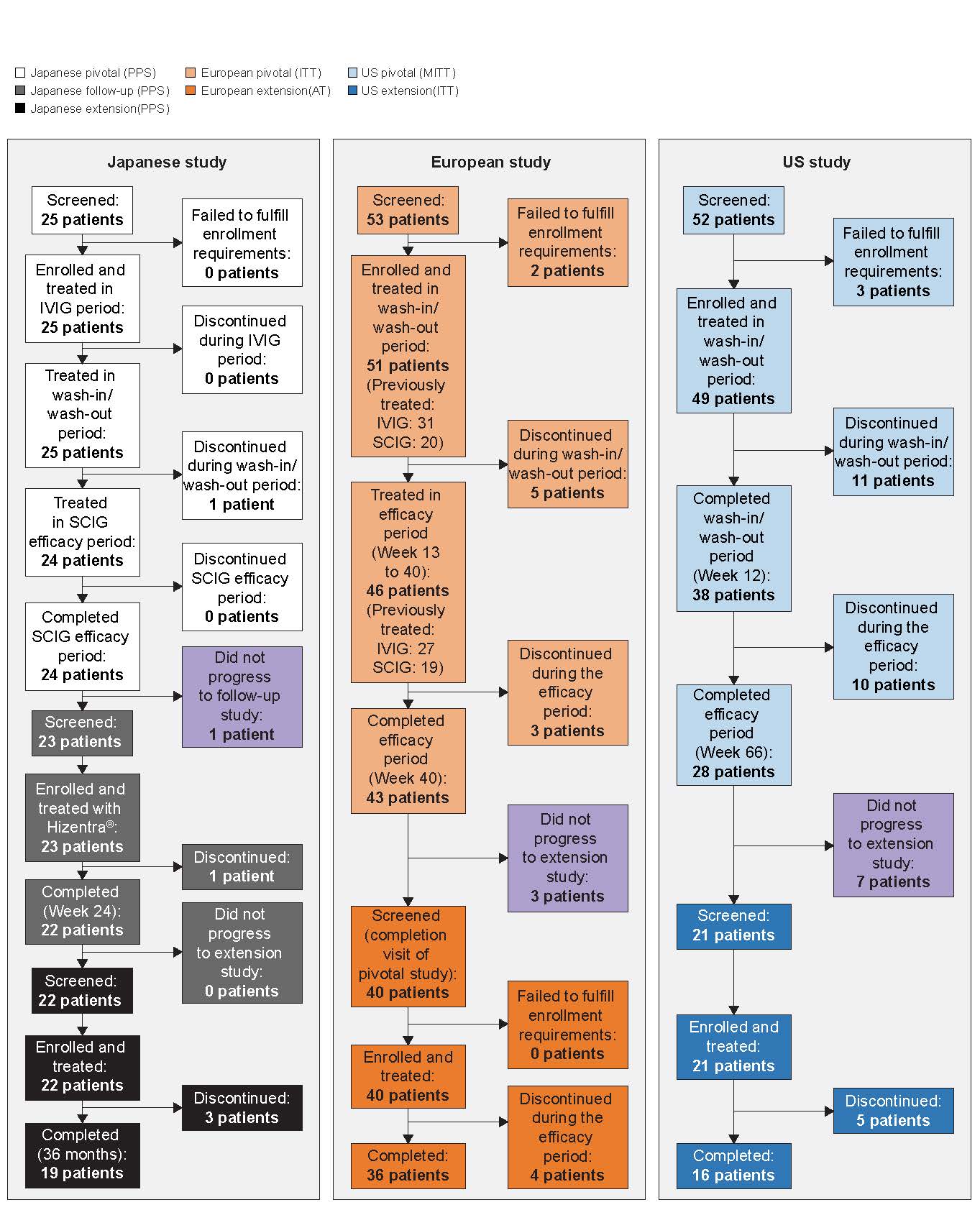


## Fig. S2


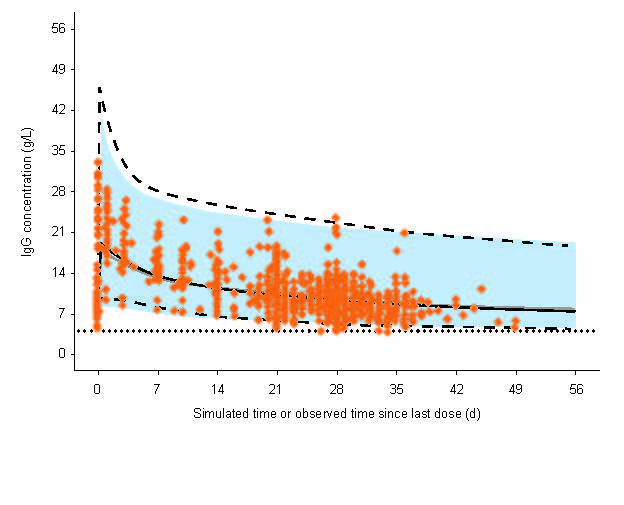

Supplement: Supplementary file 1 — (DOCX 348 kb) [file 10875_2018_560_MOESM1_ESM.docx]
